# 2-[(4-Chlorobenzyl)iminomethyl]phenol

**DOI:** 10.1107/S1600536810031508

**Published:** 2010-08-18

**Authors:** Chuttree Phurat, Thapong Teerawatananond, Nongnuj Muangsin

**Affiliations:** a Research Centre of Bioorganic Chemistry, Department of Chemistry, Faculty of, Science, Chulalongkorn University, Bangkok, 10330, Thailand

## Abstract

The title Schiff base compound, C_14_H_12_ClNO, was prepared from 4-chloro­benzyl­amine and salicyl­aldehyde. The mol­ecule is V-shaped: the dihedral angle between the aromatic rings is 67.51 (5)°. The rings are located on the opposite side of the C=N bond, giving an *E* configuration. An intra­molecular N—H⋯O hydrogen bond generates a *S*(6) ring. In the crystal structure, only weak non-classical C—H⋯O contacts are observed.

## Related literature

For background to Schiff base ligands and their biological activity, see: Adsule *et al.* (2006 ▶); Karthikeyan *et al.* (2006 ▶). For related structures, see: Tariq *et al.* (2010 ▶); Khalaji & Simpson (2009 ▶). For the graph-set analysis of hydrogen-bond patterns, see: Bernstein *et al.* (1995 ▶). For the synthesis, see: Kannappan *et al.* (2005 ▶).
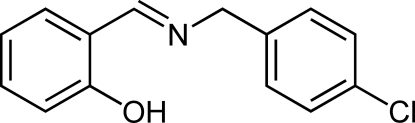


## Experimental

### 

#### Crystal data

C_14_H_12_ClNO
*M* *_r_* = 245.7
Orthorhombic, 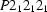

*a* = 6.2876 (2) Å
*b* = 12.2267 (3) Å
*c* = 16.2664 (5) Å
*V* = 1250.51 (6) Å^3^
*Z* = 4
Mo *K*α radiation
μ = 0.29 mm^−1^
*T* = 296 K
0.45 × 0.20 × 0.20 mm

#### Data collection

Bruker SMART APEXII CCD area-detector diffractometer
Absorption correction: multi-scan (*SADABS*; Bruker,2008 ▶) *T*_min_ = 0.933, *T*_max_ = 0.944
10586 measured reflections
1479 independent reflections
1119 reflections with *I* > 2σ(*I*)
*R*_int_ = 0.028

#### Refinement

*R*[*F* ^2^ > 2σ(*F* ^2^)] = 0.038
*wR*(*F* ^2^) = 0.107
*S* = 1.04
1479 reflections
155 parameters
143 restraints
H-atom parameters constrained
Δρ_max_ = 0.20 e Å^−3^
Δρ_min_ = −0.29 e Å^−3^

### 

Data collection: *APEX2* (Bruker, 2008 ▶); cell refinement: *SAINT* (Bruker, 2008 ▶); data reduction: *SAINT*; program(s) used to solve structure: *SHELXS97* (Sheldrick, 2008 ▶); program(s) used to refine structure: *SHELXL97* (Sheldrick, 2008 ▶); molecular graphics: *ORTEP-3* (Farrugia, 1997 ▶); software used to prepare material for publication: *SHELXL97*.

## Supplementary Material

Crystal structure: contains datablocks global, I. DOI: 10.1107/S1600536810031508/ds2046sup1.cif

Structure factors: contains datablocks I. DOI: 10.1107/S1600536810031508/ds2046Isup2.hkl

Additional supplementary materials:  crystallographic information; 3D view; checkCIF report

## Figures and Tables

**Table 1:** Hydrogen-bond geometry (Å, °)

*D*—H⋯*A*	*D*—H	H⋯*A*	*D*⋯*A*	*D*—H⋯*A*
O1—H1*A*⋯N1	0.82	1.86	2.587 (3)	147
C11—H11⋯O1^i^	0.93	2.53	3.369 (4)	150

Symmetry code: (i) 
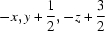
.

**Fig. 1.**
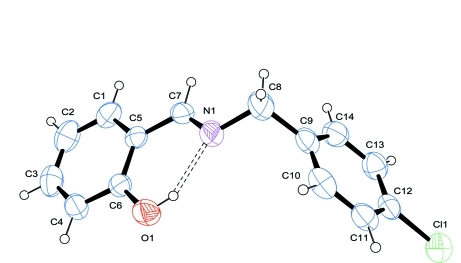
The structure of the title compound with displacement ellipsoids for the non-hydrogen atoms drawn at the 30% probability level. Hydrogen bond is shown as dashed line.

## supplementary crystallographic information

### Comment

Schiff base complexes have gained importance from physiological and pharmacological activities point of view (Adsule *et al.*, 2006). As part of our research efforts in the area of transition metal complex-based anticancer agents, the title compound has been prepared as a ligand by Schiff base reaction between 4-chlorobenzylamine and salicylaldehyde. We report herein on the crystal structure of the title compound.

The molecule adopts a *V*-shape structure. The dihedral angle between the chlorobenzene ring and 2-methyliminophenol moiety is 67.51 (5)°. The 2-methyliminophenol (C1 to C8, N1 and O1) moiety is nearly planar (r.m.s. deviation = 0.002 Å). The chlorobenzene and 2-methyliminophenol groups are located on the opposite side of the C=N bond, showing an *E* configuration. Intramolecular N—H···O hydrogen bond generates a *S*(6) ring. In the crystal structure, only weak non-classical C—H···O contact is observed.

### Experimental

The title compound was prepared according to the method reported in the literature (Kannappan *et al.*, 2005). 4-Chlorobenzylamine (2.80 ml. 2.88 g, 0.02 mol) was added to a stirred ethanol solution of salicylaldehyde (2.50 ml, 2.86 g, 0.02 mol). The reaction mixture was stirred at reflux for 1 h and then the mixture was allowed to stand at room temperature for 1 week to give yellow cystals suitable for X-ray diffraction analysis.

### Refinement

All other H-atoms were refined using a riding model with d(C—H) = 0.95 Å, *U*_iso_= 1.2*U*_eq_ (C) for aromatic and 0.98 Å, *U*_iso_ = 1.5*U*_eq_ (C) for CH_3_ H atoms. The absolute structure could not be determined and therefore 1,031 Friedel opposites were merged.

### Crystal data

**Table:**

C_14_H_12_ClNO	*F*(000) = 512
*M**_r_* = 245.7	*D*_x_ = 1.305 Mg m^−^^3^
Orthorhombic, *P*2_1_2_1_2_1_	Mo *K*α radiation, λ = 0.71073 Å
Hall symbol: P 2ac 2ab	Cell parameters from 5069 reflections
*a* = 6.2876 (2) Å	θ = 2.5–22.8°
*b* = 12.2267 (3) Å	µ = 0.29 mm^−^^1^
*c* = 16.2664 (5) Å	*T* = 296 K
*V* = 1250.51 (6) Å^3^	Prism, yellow
*Z* = 4	0.45 × 0.20 × 0.20 mm

### Data collection

**Table:**

Bruker SMART APEXII CCD area-detector diffractometer	1479 independent reflections
Radiation source: Mo Kα	1119 reflections with *I* > 2σ(*I*)
graphite	*R*_int_ = 0.028
φ and ω scans	θ_max_ = 26.3°, θ_min_ = 2.1°
Absorption correction: multi-scan (*SADABS*; Bruker,2008)	*h* = −7→7
*T*_min_ = 0.933, *T*_max_ = 0.944	*k* = −15→15
10586 measured reflections	*l* = −17→20

### Refinement

**Table:**

Refinement on *F*^2^	143 restraints
Least-squares matrix: full	H-atom parameters constrained
*R*[*F*^2^ > 2σ(*F*^2^)] = 0.038	*w* = 1/[σ^2^(*F*_o_^2^) + (0.0436*P*)^2^ + 0.2636*P*] where *P* = (*F*_o_^2^ + 2*F*_c_^2^)/3
*wR*(*F*^2^) = 0.107	(Δ/σ)_max_ < 0.001
*S* = 1.04	Δρ_max_ = 0.20 e Å^−^^3^
1479 reflections	Δρ_min_ = −0.29 e Å^−^^3^
155 parameters	

### Special details

**Table:**

Geometry. All e.s.d.'s (except the e.s.d. in the dihedral angle between two l.s. planes) are estimated using the full covariance matrix. The cell e.s.d.'s are taken into account individually in the estimation of e.s.d.'s in distances, angles and torsion angles; correlations between e.s.d.'s in cell parameters are only used when they are defined by crystal symmetry. An approximate (isotropic) treatment of cell e.s.d.'s is used for estimating e.s.d.'s involving l.s. planes.

### Fractional atomic coordinates and isotropic or equivalent isotropic displacement parameters (Å^2^)

**Table:**

	*x*	*y*	*z*	*U*_iso_*/*U*_eq_	
C1	0.7530 (5)	−0.3508 (3)	0.9530 (2)	0.0818 (9)	
H1	0.8887	−0.3216	0.9586	0.098*	
C2	0.7031 (8)	−0.4471 (3)	0.9927 (2)	0.1008 (12)	
H2	0.8045	−0.4827	1.0246	0.121*	
C3	0.5038 (8)	−0.4895 (3)	0.9849 (2)	0.0974 (10)	
H3	0.4697	−0.5542	1.0119	0.117*	
C4	0.3524 (6)	−0.4384 (2)	0.93779 (19)	0.0815 (8)	
H4	0.2173	−0.4687	0.9329	0.098*	
C5	0.6053 (4)	−0.2970 (2)	0.90492 (15)	0.0587 (6)	
C6	0.4010 (4)	−0.3415 (2)	0.89741 (17)	0.0616 (7)	
C7	0.6613 (5)	−0.1965 (2)	0.86325 (17)	0.0711 (7)	
H7	0.7986	−0.1693	0.869	0.085*	
C8	0.5987 (7)	−0.0429 (3)	0.7791 (3)	0.1127 (13)	
H8A	0.5962	−0.0524	0.7199	0.135*	
H8B	0.7433	−0.0256	0.7954	0.135*	
C9	0.4535 (6)	0.0487 (2)	0.80299 (19)	0.0789 (9)	
C10	0.2539 (7)	0.0585 (3)	0.76832 (19)	0.0857 (9)	
H10	0.2115	0.0083	0.7287	0.103*	
C11	0.1157 (5)	0.1408 (2)	0.79093 (19)	0.0802 (8)	
H11	−0.0181	0.1461	0.7669	0.096*	
C12	0.1783 (5)	0.2140 (2)	0.84889 (19)	0.0748 (8)	
C13	0.3760 (5)	0.2080 (3)	0.8837 (2)	0.0845 (9)	
H13	0.4181	0.259	0.9228	0.101*	
C14	0.5117 (5)	0.1255 (3)	0.8600 (2)	0.0877 (9)	
H14	0.6466	0.1219	0.8833	0.105*	
Cl1	0.00322 (17)	0.31716 (7)	0.87960 (8)	0.1204 (4)	
N1	0.5305 (4)	−0.14462 (19)	0.81941 (15)	0.0780 (7)	
O1	0.2507 (3)	−0.29358 (18)	0.85185 (14)	0.0864 (6)	
H1A	0.2973	−0.2364	0.8325	0.13*	

### Atomic displacement parameters (Å^2^)

**Table:**

	*U*^11^	*U*^22^	*U*^33^	*U*^12^	*U*^13^	*U*^23^
C1	0.0706 (17)	0.0856 (19)	0.089 (2)	0.0140 (17)	−0.0191 (17)	−0.0254 (16)
C2	0.122 (3)	0.093 (2)	0.087 (2)	0.033 (2)	−0.020 (2)	−0.001 (2)
C3	0.137 (3)	0.0691 (18)	0.086 (2)	0.012 (2)	0.014 (3)	0.0082 (16)
C4	0.086 (2)	0.0660 (16)	0.092 (2)	−0.0105 (17)	0.0111 (18)	−0.0036 (16)
C5	0.0583 (13)	0.0605 (13)	0.0573 (14)	0.0023 (12)	0.0008 (12)	−0.0156 (12)
C6	0.0605 (14)	0.0610 (14)	0.0632 (15)	−0.0004 (12)	−0.0037 (13)	−0.0079 (12)
C7	0.0608 (14)	0.0639 (15)	0.0888 (19)	−0.0073 (14)	0.0169 (16)	−0.0173 (14)
C8	0.129 (3)	0.0797 (19)	0.130 (3)	0.006 (2)	0.059 (3)	0.028 (2)
C9	0.091 (2)	0.0639 (16)	0.0820 (19)	−0.0101 (16)	0.0210 (18)	0.0202 (14)
C10	0.111 (2)	0.0767 (19)	0.0700 (18)	−0.0259 (19)	0.0009 (19)	0.0051 (16)
C11	0.0789 (18)	0.0806 (18)	0.0811 (19)	−0.0202 (17)	−0.0148 (17)	0.0214 (16)
C12	0.0780 (17)	0.0599 (14)	0.0864 (19)	−0.0126 (15)	0.0028 (16)	0.0179 (14)
C13	0.090 (2)	0.0751 (17)	0.089 (2)	−0.0144 (17)	−0.0140 (19)	−0.0004 (16)
C14	0.0728 (18)	0.088 (2)	0.102 (2)	−0.0088 (19)	−0.007 (2)	0.0208 (18)
Cl1	0.1050 (7)	0.0765 (5)	0.1799 (10)	0.0032 (6)	0.0167 (8)	0.0086 (6)
N1	0.0850 (16)	0.0658 (13)	0.0831 (15)	−0.0002 (14)	0.0188 (15)	0.0061 (12)
O1	0.0654 (11)	0.0876 (15)	0.1063 (16)	−0.0097 (12)	−0.0213 (12)	0.0083 (13)

### Geometric parameters (Å, °)

**Table:**

C1—C2	1.379 (5)	C8—C9	1.497 (5)
C1—C5	1.380 (4)	C8—H8A	0.97
C1—H1	0.93	C8—H8B	0.97
C2—C3	1.362 (6)	C9—C14	1.369 (4)
C2—H2	0.93	C9—C10	1.381 (5)
C3—C4	1.372 (5)	C10—C11	1.379 (5)
C3—H3	0.93	C10—H10	0.93
C4—C6	1.389 (4)	C11—C12	1.358 (4)
C4—H4	0.93	C11—H11	0.93
C5—C6	1.401 (4)	C12—C13	1.367 (4)
C5—C7	1.446 (4)	C12—Cl1	1.747 (3)
C6—O1	1.336 (3)	C13—C14	1.376 (5)
C7—N1	1.260 (3)	C13—H13	0.93
C7—H7	0.93	C14—H14	0.93
C8—N1	1.470 (4)	O1—H1A	0.82
			
C2—C1—C5	121.3 (3)	N1—C8—H8B	109.7
C2—C1—H1	119.3	C9—C8—H8B	109.7
C5—C1—H1	119.3	H8A—C8—H8B	108.2
C3—C2—C1	119.3 (3)	C14—C9—C10	117.4 (3)
C3—C2—H2	120.3	C14—C9—C8	121.8 (3)
C1—C2—H2	120.3	C10—C9—C8	120.9 (4)
C2—C3—C4	121.1 (3)	C11—C10—C9	121.8 (3)
C2—C3—H3	119.4	C11—C10—H10	119.1
C4—C3—H3	119.4	C9—C10—H10	119.1
C3—C4—C6	120.0 (3)	C12—C11—C10	118.9 (3)
C3—C4—H4	120	C12—C11—H11	120.5
C6—C4—H4	120	C10—C11—H11	120.5
C1—C5—C6	118.7 (3)	C11—C12—C13	121.0 (3)
C1—C5—C7	120.5 (3)	C11—C12—Cl1	119.4 (3)
C6—C5—C7	120.8 (3)	C13—C12—Cl1	119.5 (3)
O1—C6—C4	118.7 (3)	C12—C13—C14	119.1 (3)
O1—C6—C5	121.8 (2)	C12—C13—H13	120.4
C4—C6—C5	119.5 (3)	C14—C13—H13	120.4
N1—C7—C5	122.3 (3)	C9—C14—C13	121.8 (3)
N1—C7—H7	118.9	C9—C14—H14	119.1
C5—C7—H7	118.9	C13—C14—H14	119.1
N1—C8—C9	109.8 (3)	C7—N1—C8	119.2 (3)
N1—C8—H8A	109.7	C6—O1—H1A	109.5
C9—C8—H8A	109.7		
			
C5—C1—C2—C3	−0.4 (5)	N1—C8—C9—C10	−76.8 (4)
C1—C2—C3—C4	0.3 (5)	C14—C9—C10—C11	−1.2 (4)
C2—C3—C4—C6	−0.3 (5)	C8—C9—C10—C11	178.4 (3)
C2—C1—C5—C6	0.5 (4)	C9—C10—C11—C12	0.0 (4)
C2—C1—C5—C7	−179.5 (3)	C10—C11—C12—C13	1.1 (4)
C3—C4—C6—O1	179.9 (3)	C10—C11—C12—Cl1	−178.8 (2)
C3—C4—C6—C5	0.3 (4)	C11—C12—C13—C14	−0.8 (4)
C1—C5—C6—O1	−179.9 (2)	Cl1—C12—C13—C14	179.1 (2)
C7—C5—C6—O1	0.1 (4)	C10—C9—C14—C13	1.5 (4)
C1—C5—C6—C4	−0.4 (4)	C8—C9—C14—C13	−178.1 (3)
C7—C5—C6—C4	179.6 (2)	C12—C13—C14—C9	−0.6 (5)
C1—C5—C7—N1	−179.4 (3)	C5—C7—N1—C8	179.8 (3)
C6—C5—C7—N1	0.6 (4)	C9—C8—N1—C7	−124.3 (3)
N1—C8—C9—C14	102.8 (4)		

### Hydrogen-bond geometry (Å, °)

**Table:**

*D*—H···*A*	*D*—H	H···*A*	*D*···*A*	*D*—H···*A*
O1—H1A···N1	0.82	1.86	2.587 (3)	147.
C11—H11···O1^i^	0.93	2.53	3.369 (4)	150.

Symmetry codes: (i) −*x*, *y*+1/2, −*z*+3/2.
